# Clinical Evaluation of Efficacy of ^99m^TC -Ethambutol in Tubercular Lesion Imaging

**DOI:** 10.1155/2010/618051

**Published:** 2010-11-02

**Authors:** Namrata Singh, A. Bhatnagar

**Affiliations:** ^1^Department of Nuclear Medicine, Institute of Nuclear Medicine & Allied Sciences (INMAS), Brig. S.K. Mazumdar Marg Timarpur, New Delhi 1100054, India; ^2^Ambedkar Center for Biomedical Research (ACBR), University of Delhi, Delhi 110007, India

## Abstract

*Purpose*. The aim of this work was to develop specific radiopharmaceutical and to evaluate its efficacy in human to detect and locate the tubercular lesion. *Materials and Methods*. ^99m^Tc-Ethambutol (EMB) was produced by direct labeling method. *In vitro and in vivo* biological studies and animal experiments were done. Phase I Clinical trial was performed. As per plan, 2 normal human subjects for biodistribution studies and fourteen patients (8 males and 6 females; age range of 25–50, with one patient aged 12 years as an exception) were chosen for clinical trial. Whole body scan and spots were acquired at 1 hour and 4 hour. Angiography, blood pool, and 24-hours spot images of the infected areas were also acquired. *Result*. Radiolabeling yielded >85% of labeled complex. *In vitro and in vivo* biological studies and animal experiments indicated ^99m^Tc-EMB as a specific tuberculosis imaging agent. The biodistribution study in normal human subjects suggested stability of ^99m^Tc-EMB, with main excretory pathways being renal and hepatobiliary, which appeared to be similar to the known behavior of unlabeled EMB. No adverse reactions were observed. ^99m^Tc-EMB got localized in pulmonary and bone tubercular lesions. Scintigrams of ^99m^Tc-EMB and ^99m^Tc-Ciprofloxacin were compared at different time intervals. *Conclusion*. The present study states that developed ^99m^Tc-EMB has high potential to qualify as a specific tuberculosis imaging radiopharmaceutical and is safe for human use.

## 1. Introduction

Tuberculosis (TB) is dreaded infectious affecting almost every organ of the body and still it is one of the major health hazards with sophisticated research, high-tech drug design, and various diagnostic modalities. Rapid and accurate diagnosis ofinfected patients is a cornerstone of the global tuberculosis control strategies. Microscopic examination, Bacterial culture, Radiometric methods (BACTEC, MGIT, etc.), Chromatographic procedures, Immunodiagnostic modalities (ELISA, RIA, SAFA etc), Molecular diagnostic tools (PCR, RFLP, LCR, etc.),and Radiological and Imaging modalities have played very important roles in diagnosis of tubercular infection.Despite several advantages, these modalities have certain limitations in diagnosis of TB. Radionuclide emission-based nuclear medicine modality is a noninvasive technique, which is a quick, sensitive, and specific method to detect as well as locate the lesion at any anatomical site at early stage of the disease. A wide variety of radiopharmaceuticals are used now a day for the diagnosis of infection and inflammation. Ga-67 citrate was one of the first radiopharmaceuticals developed for infection and inflammation [1] imaging. Ga-67 citrate was used for detection of pulmonary and extrapulmonary TB but it had limitation of being nonspecific and incapable of differentiating between infection and inflammation. Nonspecific SPECT agents like ^99m^Tc-ethylene cysteine dimer have been used for diagnosis of tubercular meningitis.^99m^Tc-Tetrofosmin [2], FDG-PET [3], and ^99m^Tc-MIBI [4] were used for showed concentration in tubercular lesion by means of nonspecific uptake but these radiotracers had limited clinical success. ^99m^Tc-Ciprofloxacin (Infecton) possesses many properties to be evaluated as novel infection imaging agent [5]. ^99m^Tc-Ciprofloxacin was nonspecific and showed low sensitivity in nonbony for tubercular lesion. In view of these limitations and disadvantages, a suitable ligand, that is, specific first line antitubercular drug Ethambutol (EMB) [6] was chosen for detection as well as localization of the lesion using nuclear medicine modality. EMB was radiolabeled with Tc-99m. High labeling efficiency (>85%), *in vitro*, *in vivo* stability, biodistribution, and pharmacokinetic parameters were consistent with the original drug [7] which suggested that ^99m^Tc-EMB was safe for diagnostic use. The objectives of the work was (a) to prepare and characterize ^99m^Tc-EMB (b) to compare data obtained with ^99m^Tc-EMB in living systems with the known behavior of its unlabeled form, and (c) to conduct clinical trials and evaluate its efficacy in human to detect and locate the tubercular lesion.

## 2. Materials and Methods

### 2.1. Radiocomplexation of EMB

Ethambutol was labeled with ^99m^Tc. Radiocomplexation was standardized [6]. Briefly, 2 mg of ethambutol (sigma chemicals, USA) was taken in a vial and was dissolved in 1 ml of 0.015 M NaCl_2_ solution. To the ethambutol solution, 200 *μ*l (400 *μ*g) of 2 mg/ml solution of stannous tartarate dissolved in sterile water for the injection (to increase shelf life of the reducing agent) was added and mixed well. 50 *μ*g of *ρ*-amino benzoic acid was added as a stabilizer to the solution. To it, 75–400 MBq of freshly eluted ^99m^Tc was added using sterilized fine needle syringe. The mixture was swirled and was allowed to react for 15 minutes. Radiochemical purity was determined by ITLC method. ^99m^Tc-EMB was characterized by Infrared (IR) and Mass spectral (MS) studies. *In vivo *and* in vitro *experiments were done. Good Manufacturing Practice measures were followed strictly, using fresh chemicals and sterile laboratory ware.

### 2.2. In Vitro and In Vivo studies

*In vitro* serum and blood stability studies of ^99m^Tc-EMB were carried out. ^99m^Tc-EMB was mixed with serum and blood separately in vials (in triplicate), and samples were taken out from the vials at 1 hour, 4 hours, and 24 hours and assessed by ITLC system. Biodistribution and pharmacokinetic studies were done in Balb/c mice and New Zealand white rabbits, respectively. Parameters like plasma clearance, Plasma protein binding, *T*_1/2_, and volume of distribution (*V*_*D*_) were calculated and compared with the known values of the unlabeled EMB [7]. All tests were done in triplicate. The results were compared quantitatively and qualitatively. Experiments were carried out in compliance with the relevant national laws relating to the conduct of animal experimentation. Biological activity of ^99m^Tc-EMB was evaluated by *in vitro* pharmacological studies, that is, Minimum inhibitory concentration (MIC) experiment, drug uptake studies, and colony forming assay (CFU assay). Scintigraphy was done in New Zealand white rabbit to evaluate biodistribution of ^99m^Tc-EMB. 

### 2.3. Human Studies

#### 2.3.1. Study Design

The stated objectives of the human trial were (a) to confirm safety aspects of the new radiopharmaceutical in humans, (b) to check consistency and uniformity of behavior of the tracer in humans, (c) to suggest a practical study protocol, and (d) to study its uptake in known active and inactive tubercular lesions. As per plan, two normal human subjects and fourteen patients (with freshly diagnosed and dormant lesion) were chosen for the study.

#### 2.3.2. Patient Selection

Institutional Ethical Committee permission in accordance with the guidelines provided by Indian Council of Medical Research (ICMR) and Helsinki Declaration of 1975, as revised in 2000 and patient's consent was taken prior to initiation of the human studies. The two subjects were healthy adult male volunteers without any known disease. The other Fourteen were ambulatory patients with proven tuberculosis with known sites of infection. Four had freshly diagnosed sensitive (2) and resistant (2) pulmonary tuberculosis (diagnosed by sputum positivity and sites of infection identified by x-ray chest), eight patients had bone tuberculosis lesions (proof of infection and site identification by radiology and serology in clinical context), and two had old dormant bony lesions. A comparative study of ^99m^Tc-Ciprofloxacin and ^99m^Tc-EMB was done in a patient having lesion in acetabulum. Only those patients were recruited for the study that were not overtly sick and did not suffer from any other disease or disorder. Details of patients (age/sex) and their clinical status are shown in Table 1. The patients were admitted for at least a day for observation for precaution, though no detailed investigations were done for creating detailed toxicity data profile of ^99m^Tc-EMB.

#### 2.3.3. Scintigraphy Procedure

The patients were not required to fast before scintigraphy. 370 MBq of freshly prepared ^99m^Tc-EMB was injected intravenously as a slow bolus in adults. Angiography, blood pool phase, and spot acquisitions of sites known to harbor tubercular lesions were taken at 1 hour, 4 hours, and 24 hours in the best view using a standard dual-head Gamma Camera. Acquisition time was kept flexible and was depended upon count density of the field. Whole Body acquisitions were taken at 1 and 4 hours at slow speed (10–15 cm/min). Anterior and posterior views of abdomen and chest were acquired to aid biodistribution assessment. SPECT data was acquired in two cases with bony lesions at 4–6 hours. The patients were critically observed for any signs and symptoms and any deviation in the vitals throughout the day.

#### 2.3.4. Results

The radiochemical purity of ^99m^Tc-EMB was found to be >85%. *In vitro* stability study of ^99m^Tc-EMB in blood and serum showed that only 3%-4% of technetium leached out from the complex till 24 hours of incubation. IR spectra of unlabeled EMB gave peak at the region of *∼* 3300–2900  ^cm-1^ due to NH and OH stretch in EMB structure. The absence of peak in ^99m^Tc-EMB indicated that ^99m^Tc binds to these groups by protonation. Mass spectra of unlabeled EMB showed peak at 102.5 and 116.2 due to NH and OH groups and the absence of these peaks in ^99m^Tc-EMB indicated that ^99m^Tc binds to NH and OH groups. Organ biodistribution done in Balb/c mice showed maximum accumulation of ^99m^Tc-EMB in kidneys followed by liver and intestine shown in Table 2 [8].

Various pharmacokinetic parameters of ^99m^Tc-EMB showed similarity with its unlabeled counterpart shown in Table 3. Plasma protein binding studies showed that *∼*85% of ^99m^Tc-EMB was bound to plasma protein till 24 hours. *In vitro* pharmacological studies indicated the retention of biological activity of EMB after radiocomplexation. Scintigraphy done in New Zealand white rabbits at 1 hour and 4 hours postinjection showed high tracer uptake in the kidneys and liver. Intestinal activity was evident signifying gall bladder excretion. No activity was seen in the stomach region [6]. 

#### 2.3.5. Scintigraphy and Patient Studies

Sixteen human subjects were available for assessing safety aspects of ^99m^Tc-EMB. Two healthy individuals were chosen for studying biodistribution of ^99m^Tc-EMB and fourteen patients for studying lesion uptake pattern. No adverse effects or allergic reactions were noted in the patients in the immediate and acute periods following tracer injection. The vitals were checked periodically, which remained stable. No problem was reported in the patients kept under observation overnight. Since the drug molecule and the reducing agent (stannous tartarate) are well-known clinical entities, used in complex formation are minute diagnostic doses; detailed toxicity data were not created.

#### 2.3.6. Human Biodistribution of ^99m^Tc-EMB

**Figure 1:**
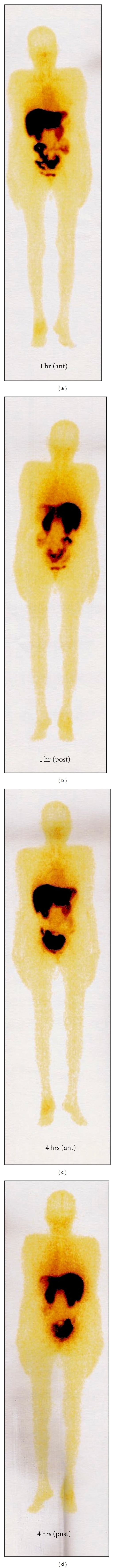
Whole body scan of human (healthy volunteer) showing biodistribution of ^99m^Tc-EMB in human at 1 hour anterior and posterior and 4 hours anterior and posterior postinjection of radiotracer.

Whole-body scans taken at 1 and 4 hours and spots at 24 hours were analyzed qualitatively. The ^99m^Tc-EMB got well distributed in the body compartments by 1 hour of postinjection (except blood-brain barrier which was never breached), with marginally higher concentration in blood as shown by faint cardiac and great veins visualization. Hepatic accumulation of radioactivity appeared to increase slowly with time, which probably reduced after 4 hours. Radioactivity was visualized in intestine before 4 hours of postinjection of ^99m^Tc-EMB with frequent gall-bladder visualization. Significant urinary activity was seen by 1 hour and kidneys were the main excretory organs. Significant renal cortex retention of ^99m^Tc-EMB was also a constant finding (Figure 1). Uptake of ^99m^Tc-EMB was fairly uniform in both lungs and appeared to buildup with time, probably stabilizing by 4 hours, which reduced at 24 hours. No uptake of ^99m^Tc-EMB was seen in dormant cases. There was no bone or bone marrow activity. Interestingly, no activity was observed in epiphysis, stomach, and thyroid (Figure 1).

#### 2.3.7. Study Protocol and Lesion Uptake

**Figure 2:**
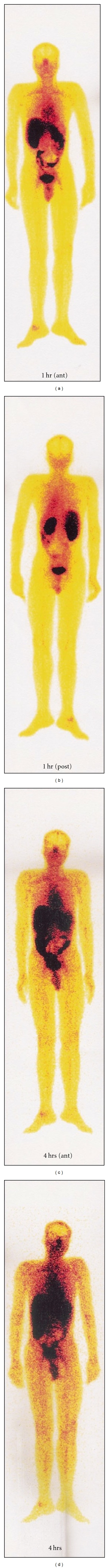
Whole body scan of human (healthy volunteer) showing biodistribution of ^99m^Tc-EMB in human at 1 hour anterior and posterior and 4 hours anterior and posterior (postinjection of radiotracer and lesion in ankle (Bone TB).

**Figure 3:**
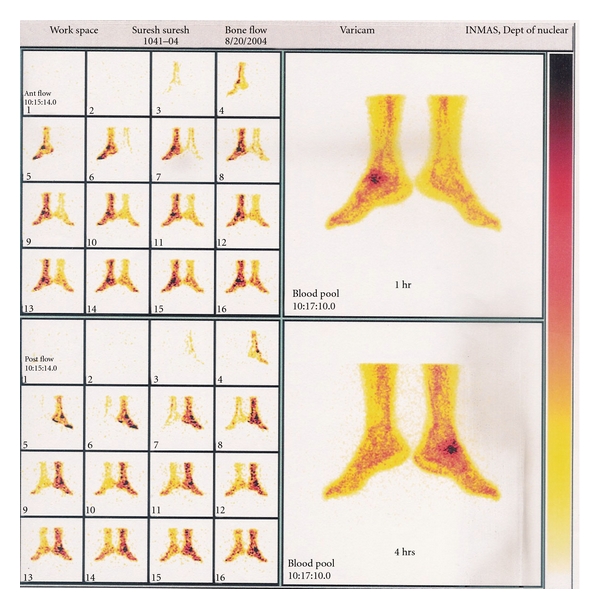
SPECT and scintigrams of patient, showing preferential localization of radiotracer in the ankle lesion after 1 hr and 4 hours postinjection of 370 MBq of ^99m^Tc-EMB.

**Figure 4:**
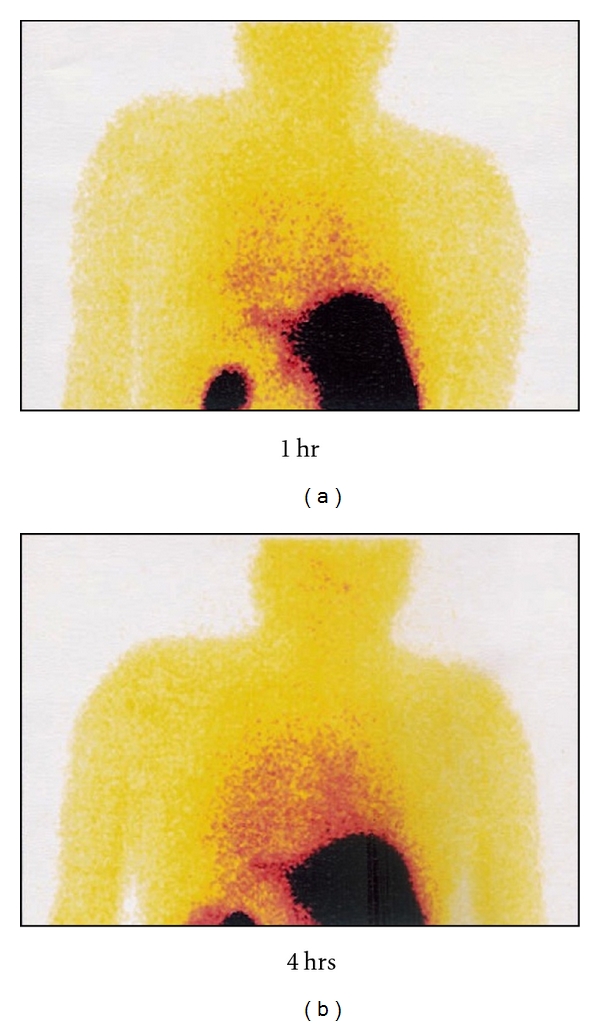
Scintigrams of patient, showing preferential localization of radiotracer in the lung lesion after 1 hour and 4 hours postinjection of 370 MBq of ^99m^Tc-EMB.

**Figure 5:**
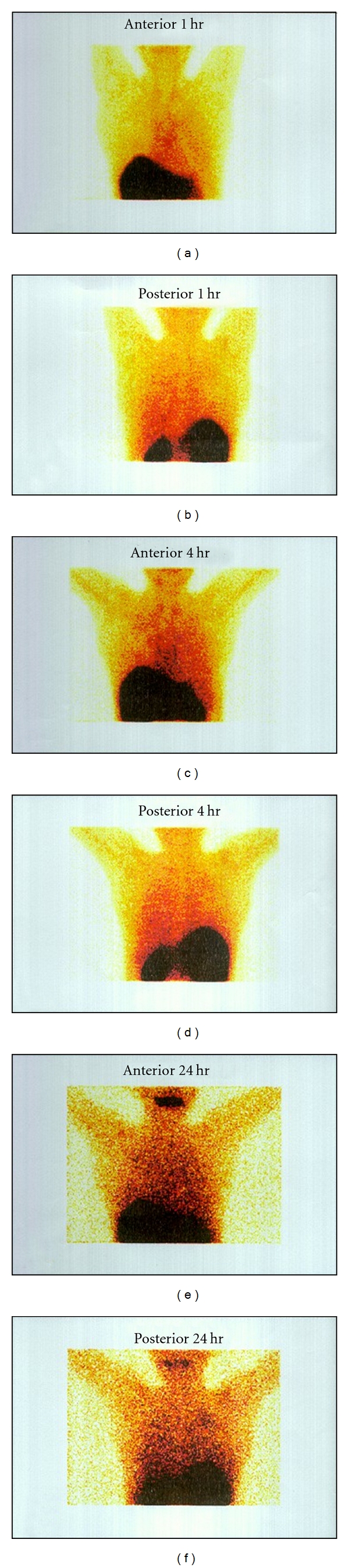
Scintigrams of human showing preferential localization of radiotracer in the resistant lung lesion after 1, 4, and 24 hours postinjection of 370 MBq of ^99m^Tc-EMB.

**Figure 6:**
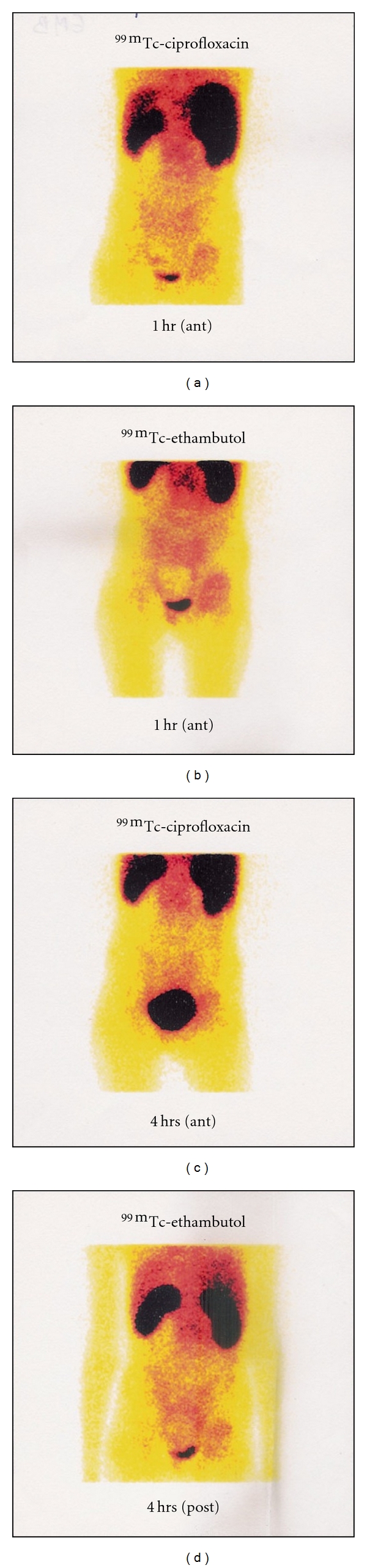
Scintigram showing comparative study of preferential localization of ^99m^Tc-Ciprofloxacin and ^99m^Tc-EMB in a patient after 1 hour (Figures 1 and 2, resp.) and 4 hours (Figures 3 and 4, resp.) postinjection of 370 MBq of ^99m^Tc-Ciprofloxacin and ^99m^Tc-EMB, respectively.

A total of fourteen subjects were identified as infected cases in prescan diagnosis (Table 1). Four had lesion in lungs (soft-tissue) and ten had bone tubercular lesions in peripheral skeleton, two being dormant ones. Soft-tissue lesions infected with tuberculosis were gained in radioactivity with time. No radioactivity was observed in dormant cases. A majority of soft-tissue lesions were visualized after 4 hours and one was visualized only at 24 hours postinjection of ^99m^Tc-EMB. Interestingly, an unsuspected pulmonary lesion was discovered on one scan that was later confirmed on a fresh X-ray chest. SPECT acquisitions done on dormant pulmonary lesions seen in planer view in two patients were of poor quality and failed to locate the lesion with any definition or contrast. Bone tubercular lesions were well visualized even on one-hour scan, and appeared to gain little activity (Figure 2). Thus there was a very slow ratio enhancement of radioactivity with time (Figure 3). In general, ^99m^Tc-EMB uptakes in bone lesions were higher quantitatively as compared to the lung lesions, which required higher count collection and delayed acquisition for proper visualization. Preferential localization of ^99m^Tc-EMB was seen in sensitive (Figure 4) and resistant (Figure 5) pulmonary lesion. The comparative study of ^99m^Tc-Ciprofloxacin and ^99m^Tc-EMB showed that ^99m^Tc-Ciprofloxacin got washed away at 4 hours and ^99m^Tc-EMB was observed in the lesion till 24 hours postinjection (Figure 6).

## 3. Discussion

Each vial contains 2 mg/ml of EMB and 200 *μ*g of stannous tartarate. Both have known safety profile. Adverse effects of EMB are rare and dose dependent. Optic neuropathy or cardio-hepatotoxicity occurs very infrequently after several months of therapeutics. The drug is freely given in pediatric age group of patients. The diagnostic dose administered to the subjects was therefore inconsequential. In clinical context, it was considered as a safe radiopharmaceutical, even in children though it should be used with abundant caution in younger age group. 

The route of excretion of ^99m^Tc-EMB is through urine, mostly in its native form. A small percentage is metabolized in liver and the end products were excreted though kidneys. Very little excretion took place through the biliary pathway. ^99m^Tc-EMB is not known to selectively collect in or interact with reticuloendothelial system and bone [9]. No accumulation of the tracer in RES and bone was an important negative observation because the fact may be advantageous in improving accuracy of detection of bony tubercular lesions with the tracer. Whole body biodistribution of ^99m^Tc-EMB was consistent with these pharmacokinetic characteristics of ethambutol, including the tendency to concentrate in lung parenchyma. Plasma protein binding, blood clearance, volume of distribution, and half-life of ^99m^Tc-EMB (Table 3) were similar to unlabeled EMB [7].

Accumulation of ^99m^Tc-EMB in lung parenchyma (similar to the native drug) was a distinct advantage because poor bacterial count, low metabolic status (of mycobacterium), and fibrotic encapsulation of tubercular lesions in the lungs result in relatively poor sensitivity of any other ^99m^Tc labeled radiotracer. ^99m^Tc-EMB would be in an advantageous position compared to other tracers because a higher physiologic concentration of the radiolabeled drug in the lung parenchyma may translate into higher uptake by mycobacterium, and therefore into higher sensitivity of detection. For this reason, delayed imaging till 24 hours appeared essential for imaging. Retention of ^99m^Tc-EMB in the lesion till 24 hours indicated that ^99m^Tc-EMB was more specific radiopharmaceutical than ^99m^Tc-Ciproflxacin for tubercular lesion imaging as ^99m^Tc-Ciproflxacin got washed away at 4 hours of postinjetion.

^99m^Tc-EMB was used in humans for tubercular imaging. The purpose of this preliminary human study was to evaluate feasibility of using the ^99m^Tc-EMB in human tubercular scintigraphy. It suggested that ^99m^Tc-EMB detects and locates active sensitive as well as resistant lesions by accumulating in the lesion in rising pattern over time.

## 4. Conclusion

The mycobacterial lesion uptake study carried out so far in humans suggested that ^99m^Tc-EMB is specific and sensitive radiopharmaceutical for sensitive as well as resistant tubercular lesion detection and localization.

## Figures and Tables

**Table 1:** Clinical details of patients and result of ^99*m*^ *T*c-EMB scintigraphy.

Sex/Age	Clinical diagnosis	Site of lesion	Treatment history	Outcome
Male/40	Pulmonary TB	Lungs	Active pulmonary tuberculosis	True positive
Male/45	Pulmonary TB	Lungs	Active pulmonary tuberculosis	True positive
Male/50	Resistant pulmonary TB	Lungs	Active resistant pulmonary tuberculosis	True positive
Female/42	Resistant Pulmonary TB	Lungs	Active resistant pulmonary tuberculosis	True positive
Female/27	Bone TB	Spine	1. Old treated case of Pott's spine 2. local tenderness present	True negative
Male/43	Bone TB	Spine	Old treated case of Pott's spine.	True negative
Male/30	Bone TB	Ankle	Tuberculosis of ankle	True positive
Female/36	Bone TB	Ankle	Tuberculosis of ankle, active on MRI clinically	True positive
Male/34	Bone TB	Spine	1. Carries retropharyngeal abscesses. 2. MRI carries yellow marrow replacement in vertebral bodies.	True positive
Female/40	Bone TB	Ankle	Tuberculosis of ankle, positive in 4th metatarsal of the right foot.	True positive
Female/35	Bone TB	Lymph nodes in the neck	Tuberculosis in throat No ATT	True Positive
Male/32	Bone TB	Spine	A case of cervical TB	True positive
Male/51	Bone TB	Spine	A case of Pott's spine with local tenderness	True positive
Female/12	Bone TB	Spine and Acetabulum	1. Active Pott's spine 2. Large psoas abscess 3. Unsuspected pelvic infection later confirmed by MRI	True positive

**Table 2:** Biodistribution data of ^99*m*^ *T*c-EMB in Balb/c mice. The mice were administered 40 KBq of ^99*m*^ *T*c-INH, and the radioactivity in different organs was measured at 1 hr, 4 hrs, and 24 hrs, respectively. each value is the mean ± SD of three mice and expressed as percentage administered dose per gram organ.

Organ/Tissue	1 hour	4 hours	24 hours
Blood	2.0 ± 0.03	1.3 ± 0.19	0.8 ± 0.02
Heart	0.73 ± 0.21	0.54 ± 0.07	0.36 ± 0.6
Lungs	2.1 ± 0.18	1.88 ± 0.3	1.05 ± 0.04
Liver	5.5 ± 0.5	7.0 ± 0	3.83 ± 0.21
Spleen	1.41 ± 0.09	1.78 ± 0.43	1.04 ± 0.02
Intestine	6.21 ± 0.4	6.99 ± 0.17	2.09 ± 0.03
Kidney	10.3 ± 0.42	11.35 ± 0.32	2.02 ± 0.04
Muscle	1.6 ± 0.2	1.0 ± 0.2	1.68 ± 1.1
Bone	1.08 ± 0.28	0.88 ± 1	0.64 ± 0.05
Brain	0.165 ± 0.06	0.07 ± 0.19	0.06 ± 0.2
Stomach	1.01 ± 0.31	0.8 ± 1	1.0 ± 0.3

**Table 3:** Comparison of pharmacokinetic parameters of ^99*m*^ *T*c-EMB and unlabeled EMB.

^99m^Tc-EMB	Unlabeled ^99m^Tc-EMB
T_1/2_ = 9.5 hours	T_1/2_ = 10–15 hours
*V*_*d*_ = 2.8 L/Kg	*V*_*d*_ = 3.1 L/Kg
Cl = 16.5 ml/hr	Cl = 15–20 ml/hr
Plasma protein binding = 60%	Plasma protein binding = 10%–40%

Where, *T*_1/2_ is half-life, *V*_*d*_ is volume of distribution, and Cl is clearance.
